# The global prevalence of maternal near miss: a systematic review and meta-analysis

**DOI:** 10.15171/hpp.2019.35

**Published:** 2019-10-24

**Authors:** Sedigheh Abdollahpour, Hamid Heidarian Miri, Talat Khadivzadeh

**Affiliations:** ^1^Nursing and Midwifery Care Research Center, Mashhad University of Medical Sciences, Mashhad, Iran; ^2^Social Determinants of Health Research Center, Mashhad University of Medical Sciences, Mashhad, Iran

**Keywords:** Maternal near miss, Maternal health, Prevalence, Systematic review, Meta-analysis

## Abstract

**Background:** Improving the maternal health is one of the world’s most challenging problems. Despite significant movements over the past decades, maternal health has been still considered as a central goal for sustainable development. Maternal near miss (MNM) cases experience long-term physical and psychological effects. To present a clear portrait of the current situation, we performed a systematic review and meta-analysis with the purpose to assess the worldwide prevalence of MNM.

**Methods:** We conducted a systematic review on PubMed, Scopus and Web of Science electronic databases to find published papers in English, before March 2019 and regardless of the type of study. We, then, assessed the prevalence of MNM according to the World Health Organization(WHO) criteria. Finally, 49 papers were included in the study. Random effects meta-analysis was used to pool the available prevalence. The quality of studies was also evaluated.

**Results:** The weighted pooled worldwide prevalence of MNM, was 18.67/1000 (95% CI: 16.28-21.06). Heterogeneity was explored using subgroup analyses based on the continent and the country. We used meta-regression of MNM on MD which resulted in adjusted R-squared as78.88%.

**Conclusion:** The prevalence of MNM was considerable. Low- and middle-income countries should develop systematic approaches to improve quality of care in the facilities and to reducethe risk of MNM events, with the hope to women’s health.

## Introduction

In the way of reaching Millennium Development Goals to end preventable maternal mortality, it is still regarded as an unfinished agenda and one of the world’s most challenging problems. Despite significant movements over the past years, it still continued to be as an open discussion in the post-2015 framework for sustainable development targets and investment priority.^1^ According to the perspective of World Health Organization (WHO), every pregnant woman and newborn should receive qual­ity care throughout pregnancy, childbirth and the postnatal periods.^1^ Although there was a 45% decrease in the worldwide maternal deaths (MD) since 1990, there are still 800 deaths per day from highly preventable causes before, during, and after the time of childbirth.^1^

In 2004, WHO published a manual under the title “Beyond the Numbers: reviewing maternal death and complications to make pregnancy safer”,that emphasizes the need for going beyond merely counting deaths to developing an understanding of why they happen and how they can be prevented.^2^ In recent years the term “near miss” emerged in the lexicon of maternal health for the evaluation of the quality of obstetric care.^2^ In April 2009, a paper published by the WHO working group defined maternal near miss (MNM) as “a woman who nearly died but survived a complication that occurred during pregnancy, childbirth or within 42 days after childbirth”.^3^ In other word, a woman is considered a near miss case if she survives a life-threatening condition (i.e. organ dysfunction).^3^ Accordingly, prevalence of near-miss, MNM, started to gradually find its way as a useful measurement for the evaluation of safe motherhood programs in populations.^2^

Then, health professionals and administrators have used MNM to evaluate the facilities for maternal care to reduce the fatality among women with near miss complications and to provide a basis on which further researches are conducted.^2^ Based on WHO criteria MNM ratio (MNMR) mentions to the number of maternal near-miss cases per 1000 live births (LB).^3^ This criterion is being reviewed to improve the quality of care, because a large number of MNM cases will experience long-term physical and psychological effects.^3^ That due to the lack of accurate statistics of psychological complications and burden of disease, implementation of supportive care, screening of traumatic childbirth, and early counseling prior to the beginning of post-traumatic stress or depression was suggested.^4,5^ But at present, studies that have examined the global MNM rate are very low or old^6,7^ and according to WHO criteria, accurate statistics are not available from these mothers. Considering this gap and need for aggregated information for policy and decision making on reducing maternal morbidity and mortalities, the purpose of this study is to systematically review worldwide prevalence of MNM.

## Material and Methods

Based on the Preferred Reporting Items for Systematic Reviews and Meta-analyses (PRISMA) checklist,^8^ this study was designed to review the body of the available literature on MNM published until March 2019. We have searched the electronic databases including PubMed, Scopus and Web of Science and manually checked references of the identified relevant papers. Regarding eligibility criteria and regardless of the type of study, we include articles that have identified mothers according to WHO criteria (Including renal, cardiovascular, respiratory, hepatic, coagulation/hematological, uterine dysfunction and neurological) ^3^ as well as articles that extract the number of MD and the number of live births. We excluded studies targeted certain groups or conditions such as some ethnic groups, twin pregnancy, post-partum hemorrhage, preeclampsia, ectopic pregnancy etc because of lack of generalizability.

We restricted the search to published articles in English language. The search strategy focused on three key words or phrases: (“maternal near miss” AND (prevalence or incidence)). The full-text articles were read to confirm eligibility and to collect relevant information from the selected abstracts. Each article was read by at least two authors independently for the reading and synthesis stages. The disagreements between them were solved by consensus. The reasons for exclusion criteria were listed in the PRISMA flowchart.

The information of studies include author, year, country, continent , design of study, sample size, MNM, MD, MNMR and quality score were collected in prepared a separate page and are summarized in Table 1.^9-57^ Quality of the studies was evaluated according to the set of criteria based on Joanna Briggs Institute (JBI) guidance on conducting prevalence and incidence reviews.^58^ The quality of a paper was assessed based on criteria such as the appropriate recruitment of the participants, the representativeness of the sample, the sufficiency of the coverage of the identified sample by the data analysis, the adequacy of the sample size, the objectivity and standard of the criteria used for measurement of the condition, the detailed description of the participants and the setting, the reliability of the condition measurement, the adequacy of the response rate the appropriateness of the statistical analysis, and the appropriate coping with the low response rate, in case it existed. All the article met the high-quality standard. When the indicators were not mentioned, but contained the necessary information for its calculation, it was assessed and added to the results in the review. Among the results, the following quantitative indicators were extracted.

To explore MNM ratio with WHO approach, the number of cases in a thousand LB was considered.^3^ We applied a random effects meta-analysis to obtain the weighted average prevalence with 95% CIs for studies. Heterogeneity was estimated using the Cochran I^2^ statistic and its *P* value.^59^ Subgroup analysis was accomplished to analyze MNM as WHO criteria. All statistical analyses were conducted using Stata version 14.1(Stata Corp, College Station, TX).

## Results

**Figure 1:**
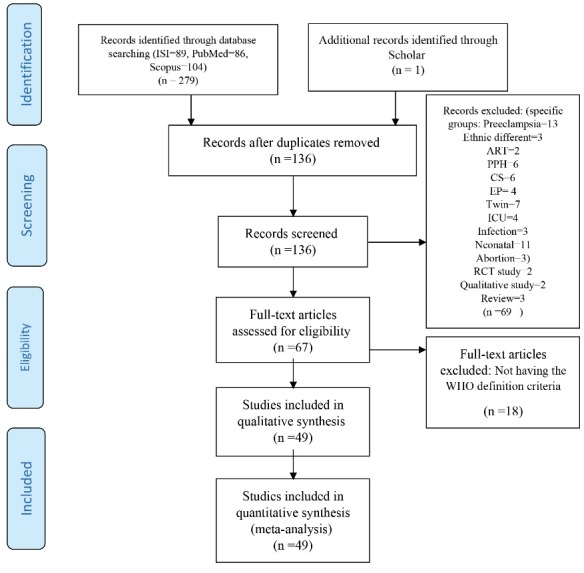
PRISMA diagram for the selection process of the articles.

The primary search recognized 300 published papers, including 86 in PubMed, 89 in Web of Science and 104 in Scopus. From those, 164 articles were elimi­nated after removal of duplicates. Nearly 135 articles were initially screened by abstract. After excluding those who had inclusion criteria, 67 articles remained for full-text screening. Out of these 67 articles, in the next step 18 were excluded either because they had not followed the WHO definition criteria. Data have been extracted from 49 articles (Figure 1). There was well agreement (88.37%) between reviewers on the terminal articles eligible for inclusion.

Depending on the study design, the selected papers were cross-sectional (n = 34), cohorts (n = 12), case control (n = 3). In almost all continents, the prevalence of MNM was investigated. No studies were conducted in North America and Australia. The number of articles conducted in Asia, South America, Africa, Europe and Oceania was 20, 9, 16, 2, and 2, respectively. Most of these studies were conducted in Brazil (n = 7) and India (n = 9). Of the 34 articles, both MNM and MD were extracted; out of 15, only the prevalence of MNM was extracted. All articles were conducted since 2012, because the WHO criteria for identifying MNM was announced this year. Prevalence of MNM is reported by all the studies ranged from 2.2 to 287.7 /1000 LB.

**Figure 2:**
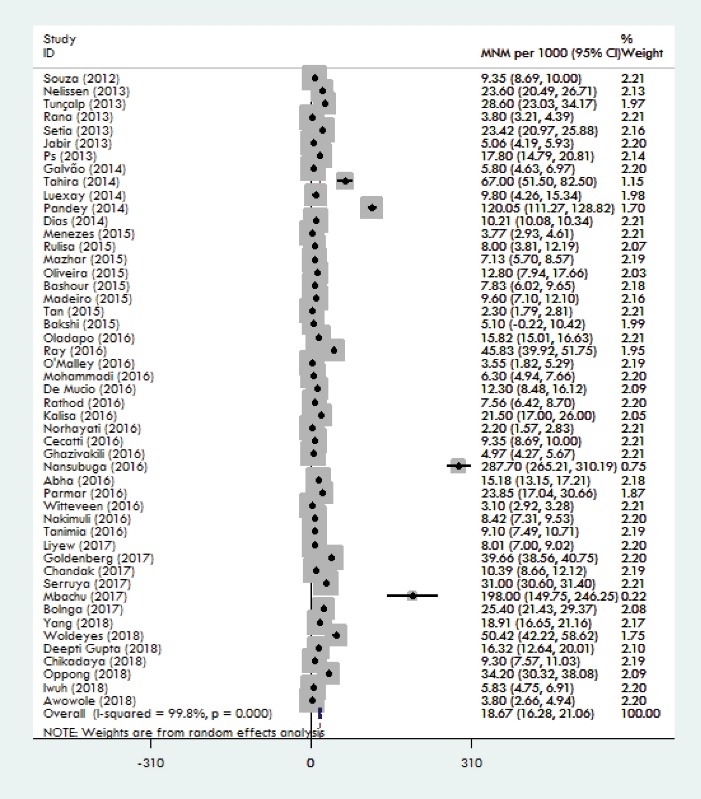
Forest plot of the pooling of overall worldwide prevalence of MNM.

**Figure 3:**
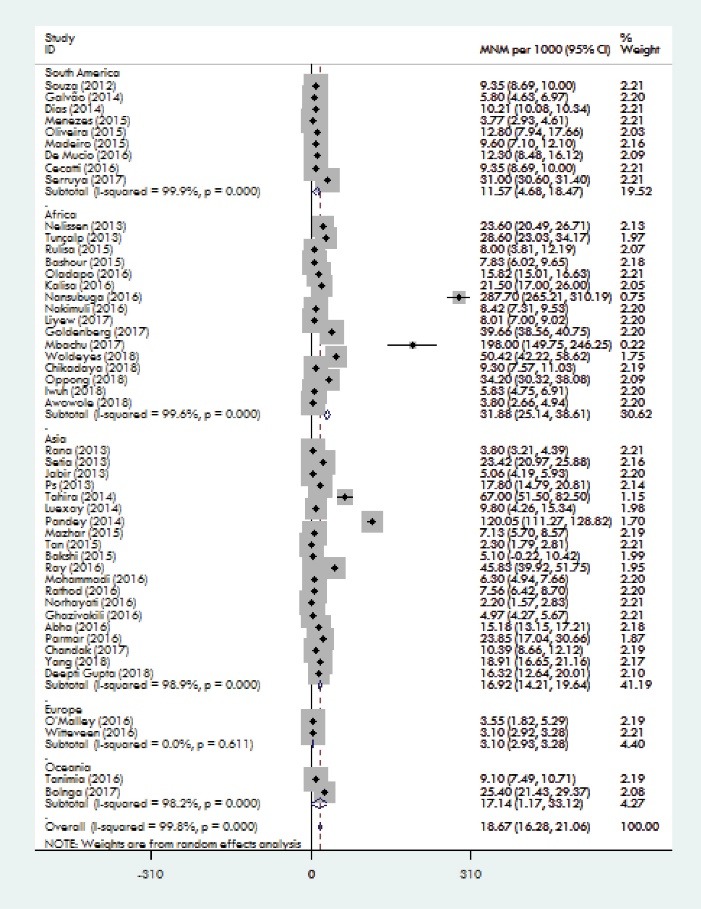
Forest plot of the pooling of overall continents prevalence of MNM.

**Figure 4:**
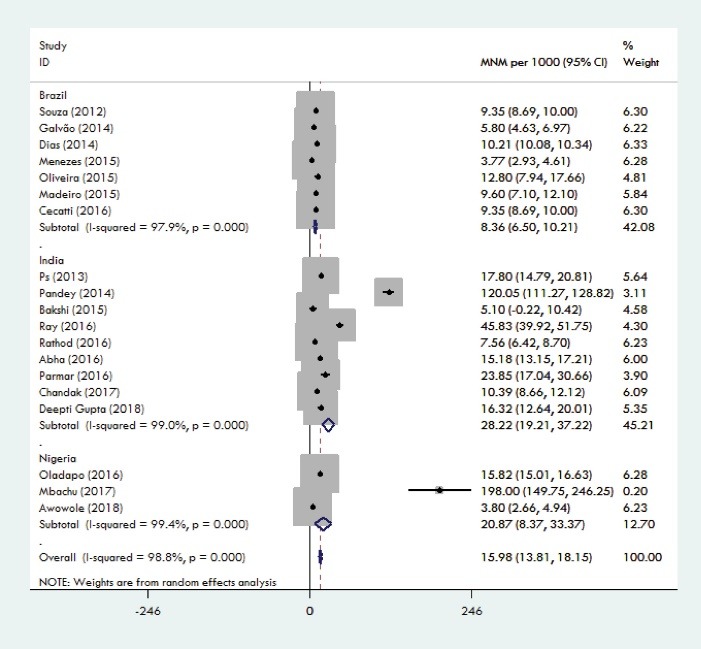
Forest plot of the pooling of overall countries prevalence of MNM.

The weighted pooled worldwide prevalence of MNM, was 18.67/1000 (95% CI: 16.28-21.06). There was a large volume of heterogeneity in the prevalence of MNM (I^2^ = 99.8%; Cochran Q-statistic *P* < 0.0001; Figure 2). The subgroup analysis will help to explore heterogeneity in the MNM prevalence. Subgroup analysis was conducted based on the continent and the country. Because maternal health indicators are very diverse in countries and continents. By continents, MNM prevalence ranged from 3.10/1000 in the Europe to 31.88/1000 LB in the Africa. Among the studies that had been conducted in Asia, weighted pooled prevalence of MNM was 16.92 (95% CI: 14.21-19.64) with significant heterogeneity between studies (I^2^= 98.9%; Cochran Q-statistic *P* < 0.001).Among the studies that had been conducted in South America, weighted pooled prevalence of MNM was 11.57 (95% CI: 4.68-18.47) with significant heterogeneity between studies (I^2^ = 99.9%; Cochran Q-statistic *P* < 0.001).Among the studies that had been conducted in Africa, weighted pooled prevalence of MNM was 31.88 [95% CI: 25.14-38.61] with significant heterogeneity between studies (I^2^ = 99.6%; Cochran Q-statistic *P* = 0.00). Among the studies that had been conducted in Europe, weighted pooled prevalence of MNM was 3.10 (95% CI: 2.93-3.28) with non-significant little heterogeneity between studies (I^2^ = 0.0%; Cochran Q-statistic *P* = 0.61). Among the studies that had been conducted in Oceania, weighted pooled prevalence of MNM was 17.14 (95% CI: 1.17-33.12) with significant heterogeneity between studies (I^2^ = 98.2%; Cochran Q-statistic *P* < 0.001; Figure 3). For heterogeneous justification, a subgroup analysis was also conducted based on the country; for example, the prevalence of MNM in Brazil was 8.36 (95% CI: 6.50-10.21) with significant heterogeneity between studies (I^2^ = 97.9%; Cochran Q-statistic *P* < 0.001). Among the studies that had been conducted in India, weighted pooled prevalence of MNM was 28.22 [95% CI: 19.21-37.22] with significant heterogeneity between studies (I^2^ = 99 %; Cochran Q-statistic *P* < 0.001) and in the Nigeria was 20.87 [95% CI: 8.37-33.37] with significant heterogeneity between studies (I^2^ = 99.4 %; Cochran Q-statistic *P* < 0.001; Figure 4). The results indicate that the heterogeneity between studies is significant in a country (Including Brazil, India and Nigeria).

To further assess the source of heterogeneity we used meta-regression of MNM on MD which resulted in adjusted R-squared as 78.88%. This means that much of the observed heterogeneity among studies could be explained by maternal death as Pearson correlation coefficient between MNM and MD among included studies was also 70.16% (*P* < 0.001). The coefficient of meta-regression shows that for each unit increase in MD (per 100 000) the average of MDM (per 1000) is expected to increase by 0.024. This implies that in order to reduce the risk of MNM events, it’s helpful to work on the risk factors on MD. The weighted pooled worldwide prevalence of MD among 35 studies that had reported it, was 294.75/100 000 LB [95% CI: 244.06-345.44]. A sensitivity analysis using *metainf* command which was performed to see whether the total result is affected by any individual studies showed that some studies had some impact on the total result but this impact was not statistically significant. Publication bias was assessed visually and statistically using funnel plot and Egger’s and Begg’s test. Regarding the asymmetry shape of the funnel plot and the *P* values of Egger’s and Begg’s test that were 0.160 and 0.000 respectively, it seems that there could be some sort of publication bias. However, the results tended to be unchanged after applying Trim and Fill approach.

## Discussion

We found that more than 18.67/1000 LB of the general population of the world suffered from MNM based on WHO criteria. So far, two systematic review in 2004^6^ and 2011^7^ have been conducted to determine the prevalence of maternal mortality globally. In recent years, a review study conducted to determine the death of mothers based on WHO criteria, has not been carried out. Therefore, comparing the prevalence’s changes during the passage of time is relatively difficult. In a review conducted by Say et al, prevalence’s of MNM ranged from 0.80% to 8.23% in studies which had disease-specific criteria while it was from 0.38% to1.09% in studies which had organ-system based criteria and it was from 0.01% to 2.99% in studies which had management-based criteria.^6^

In another review conducted by Tunçalp et al, the prevalence’s varied between 0.6 and 14.98% in studies which had disease specific criteria while it varied between 0.14% and 0.92% in studies which had Mantel (organ dysfunction) criteria and it varied between 0.04% and 4.54% in studies which had management-based criteria. A meta-analysis in the aforementioned study, estimated that MNM was 0.42% (95% CI 0.40-0.44%) for the Mantel (organ dysfunction) criteria.^7^ In both of these studies, the reported MNM prevalence is in 100 live birth, but in our study, according to the WHO definition, the number of MNM reported in 1000 live births, Which indicates that the MNM rate has declined in general, and the WHO’s unit case-identification criteria of MNM and its declare to all countries has played a major role in improving quality care.

Other results of our study were exploring the MNM prevalence in countries. In our study the prevalence of MNM according to the WHO criteria in Brazil was 8.36/1000, which is consistent with the systematic review of da Silva et al in Brazil (the MNMR varied from 4.4/1000 LB).^60^ The reason for the little statistical difference is the difference in the years of the study as well as various definitions in identifying the cases of maternal death. In the present study the MNM prevalence in middle and low income countries of Asia and Africa to compare to high-income countries, are higher; which is in line with the study of Tunçalp et al.^7^ Of course, in these continents, countries such as Iran have a MNM prevalence (1/1000 LB) similar to that of European countries.^61^ The meta-regression between MNM and MD justifies the heterogeneity of studies in countries, because even in one country, there is variation in the factors like the quality of care which are major determinant for both MD and MNM. In fact, the high correlation between MNM and MD is because of possible common risk factors.

The limitation of this study is the selection of articles based on WHO criteria that led to automatically deletion of the articles published before 2012. Despite this limitation, the study has a number of strengths. First, it is the first worldwide systematic review of the prevalence of MNM conducted according to WHO criteriathat we examined study quality, publication bias and sensitivity analysis. Secondly, the number of articles that have been Meta-analyzes is high and reflects a comprehensive view of the health of mothers.

## Conclusion

The prevalence of MNM is considerable in many countries, especially in low- and middle-income countries. An important finding of this study was, the significant and direct relationship between MNM and maternal death. This means that fluctuations in one lead to another change. Therefore, the maternal mortality index can be considered as the most reliable indicator in the assessment of maternal health. Based on this index, countries should establish a national health care system for increase standard and safety practices of health providers. It is recommended that, in view of the fact that improving maternal health is one of the goals of sustainable development by 2030, similar studies will be undertaken in the future to investigate the trend of MNM rate in order to plan the necessary program and make the appropriate policies.

## Ethical approval

Not applicable.

## Competing interests

The authors declare that they have no competing interests.

## Funding

This research received no grant from any funding agency in the public.

## Authors’ contributions

TK and SA designed the research, conducted systematic research, extracted data and wrote the manuscript. HHM and SA analyzed data. All authors had primary responsibility for the final content of the manuscript and all authors read and approved the final manuscript.

## Acknowledgements

The authors would like to extend their deepest thanks to all librarians who helped them to access information resources in Mashhad University of Medical Sciences.

**Table 1:** Specifications of studies about prevalence Maternal Near Miss based on the WHO approach in world

**Author**	**Year**	**Country**	**Continent**	**Design**	**Sample (LB)**	**MNM**	**MD**	**MNM/1000**	**JBI Score**
Souza^9^	2012	Brazil	South America	Cross-sectional	82 388	770	140	9/34	17
Jabir^10^	2013	Iraq	Asia	Cross-sectional	25 472	129	16	5/06	16
Nelissen^11^	2013	Tanzania	Africa	Cross-sectional	9152	216	32	23/6	17
Ps^12^	2013	India	Asia	Cross-sectional	7390	131		17/80	18
Rana^13^	2013	Nepal	Asia	Cohort	41 676	157		3/80	15
Tunçalp^14^	2013	Ghana	Africa	Cohort	3438	94	37	28/60	17
Setia^15^	2013	Indonesia	Asia	Cross-sectional	14 559	341		23/42	16
Dias^16^	2014	Brazil	South America	Cross-sectional	2 300 000			10/21	16
Galvão^17^	2014	Brazil	South America	Cross-sectional	16243	77	17	5/80	15
Luexay^18^	2014	Laos	Asia	Cohort	1215	11	2	9/80	15
Pandey^19^	2014	India	Asia	Case-control	5273	633	247	120/04	14
Tahira^20^	2014	Pakistan	Asia	Cross-sectional	1000	67		67	17
Bakshi^21^	2015	India	Asia	Cross-sectional	688	51	10	5/12	18
Bashour^22^	2015	Egypt*	Africa	Cross-sectional	9063	71	6	7/83	17
Madeiro^23^	2015	Brazil	South America	Cohort	5841	56	10	9/60	18
Mazhar^24^	2015	Pakistan	Asia	Cross-sectional	13 175	94	38	7/13	15
Menezes^25^	2015	Brazil	South America	Cross-sectional	20 435	77	17	3/76	16
Oliveira^26^	2015	Brazil	South America	Cross-sectional	2055	.		12/8	17
Rulisa^27^	2015	Rwanda	Africa	Cross-sectional	1739	13		8	14
Tan^28^	2015	China	Asia	Cross-sectional	34 547	8	5	2/3	15
Abha^29^	2016	India	Asia	Cohort	13 895	211	102	15/18	17
Cecatti^20^	2016	Brazil	South America	Cross-sectional	82 388	770	140	9/34	17
De Mucio^31^	2016	Latin America	South America	Cross-sectional	3196	37		12/3	16
Ghazivakili^32^	2016	Iran	Asia	Cross-sectional	38 663	192	7	4/97	16
Kalisa^33^	2016	Rwanda	Africa	Cohort	3994	86	13	21/51	16
Mohammadi^34^	2016	Iran	Asia	Case-control	12 965	82	12	6/30	15
Nakimuli^35^	2016	Uganda	Africa	Cohort	25 840	695	130	8/42	15
Nansubuga^36^	2016	Uganda	Africa	Cross-sectional	1557	434		287/70	18
Norhayati^37^	2016	Malaysia	Asia	Cross-sectional	21 579	395	2	2/20	15
Oladapo^38^	2016	Nigeria	Africa	Cross-sectional	91 724	1451	998	15/81	16
O'Malley^39^	2016	Ireland	Europe	Cross-sectional	4502	16	0	3/55	17
Parmar^40^	2016	India	Asia	Cross-sectional	1929	46	18	23/85	18
Rathod^41^	2016	India	Asia	Cohort	21 992	161	66	7/56	17
Ray^42^	2016	India	Asia	Cross-sectional	4800	220	17	45/83	16
Tanimia^43^	2016	Papua New Guinea	Oceania	Cross-sectional	13 338	122	9	9/1	16
Witteveen^44^	2016	Netherlands	Europe	Cross-sectional	371 623	1179		3/17	15
Bolnga^45^	2017	Papua New Guinea	Oceania	Cohort	6019	153	10	25/4	16
Chandak^46^	2017	India	Asia	Cross-sectional	13 186	137		10/38	16
Goldenberg^47^	2017	Zambia**	Africa	Cross-sectional	122 707	4866		39/65	15
Liyew^48^	2017	Ethiopia	Africa	Cross-sectional	29 697	238		8/01	15
Mbachu^49^	2017	Nigeria	Africa	Cross-sectional	262	52	5	198	15
Serruya^50^	2017	Latin America	South America	Cross-sectional	712 081	21985	1028	31/50	16
Awowole^51^	2018	Nigeria	Africa	Case-control	11 242	.		3/8	15
Chikadaya^52^	2018	Zimbabwe	Africa	Cohort	11 871	110	13	9/3	16
Iwuh^53^	2018	South Africa	Africa	Case-control	19 222	112	13	5/83	17
Oppong^54^	2018	Ghana	Africa	Cross-sectional	8433	288	62	34/2	17
Woldeyes^55^	2018	Ethiopia	Africa	Cross-sectional	2737	138	24	50/42	16
Yang^56^	2018	China	Asia	Cohort	14 014	265		18/90	17
Deepti Gupta^57^	2018	India	Asia	Cohort	4533	74	15	16/32	16
